# The Suffrage and Florence Nightingale

**Published:** 1917-10-20

**Authors:** 


### THE MATRONS' AND SISTERS' DEPARTMENT.

### THE SUFFRAGE AND FLORENCE NIGHTINGALE.

When any human being has stood for us as guide, philosopher, and—still more vitally—as example of thought and action, it is inevitable that when confronted by pressing questions we should ask what the opinion and what the action of such a person would be regarding the new problems or new forms of the eternal problems which we must solve. The question of the old-fashioned, simple Christian, What would Jesus do? has meant for multitudes a life deliberately planned in accordance with the answer. Those who have made life sublime have secrets of their process to communicate. "Tell me the secret of your life," said someone to Charles Kingsley, "that I may make mine beautiful too." To all who follow the profession she created such an inquiry may well direct itself towards Florence Nightingale, the woman who saw deeper into the heart of things than most men and women of her time, and acted on what she saw. Therefore she lifted not only one profession but the whole work and position of women to a higher level throughout the whole Empire and beyond it. Now the trenches that bristled with opposition to women's progress are levelled, entanglements are cut one by one; it seems certain that very soon any and every field of industry and dominion open to men will be free to a woman also, *if she is equally competent*. Here, indeed, is the crux. We cannot fail to notice that a large number of advertised offers of employment contain the words "University woman preferred."

Why? Well, presumably because that indefinable something which, when we find it in a woman, we are apt to call "masculine" strength of character," seems to be developed by a sound education. Such qualities as breadth of view, sane and sound judgment, appreciation of divergent opinions, power to master facts and reason from them, knowledge of the past that moulds foresight, and a disciplined habit of effort are more needed in the practical affairs of life than the quality of intuition which is too easily supposed capable of replacing them. Florence Nightingale had not the advantage of a University education. But the desire for mental discipline, which even reached at times to the point of self-torture, was wakened in her by the very consciousness of a call to be a saviour that came to her as a child, and was constantly repeated till the great opportunity came, and found her prepared. All through her youth bitter self-reproach for slothful and irregular habits recurs in her memoranda. In a girlhood rich in home luxury, in opportunities of travel and the pleasures of good society, with a devoted sister and parents of high intelligence, and with a decided sense of kindly duty to others less fortunate, she pined for "a life of obscurity and toil. Such a life would allow whatever I may have received of God to ripen. . . . People are in too much of a hurry to produce, and consume themselves." It is only in retirement, in silence, in meditation that are formed the men who are called to exercise an influence on society. The Church gave no help, sending her, as she felt, to do crochet in her mother's drawing-room or look well at the head of a husband's table. In truth, the discipline was there. Not merely did mother and sister preach down her heart; it was the father who first, though slowly, came to understand that the nest's uncomfortable nursling was so because it was swan and not duckling. The whole young-lady life was impossible, and she saw no other available. "Pursuing an aim not to be found in life is true misery."

It would seem to be by some such process, steel tempered by being brought to a white-heat of enthusiasm and then plunged into chill waters of disappointment and hindrance, that great characters are formed. Moses pampered in court life, then solitary in the humble life of a shepherd, had grown old before opportunity came. Rebuff, disappointment, long delay seem often to have prepared men and women for high service. The reflection comes with special force at this moment, when women are claiming that new powers of direct influence in national affairs are essential to the fulfilment of their share in national service, and the burden of the franchise will soon be laid alike on willing and unwilling shoulders. At John Stuart Mill's invitation, Miss Nightingale joined the one suffrage society that existed in 1868, and in 1871 allowed her name to be added to the General Committee list. But she did not warmly espouse the cause. "It will be years before you obtain the suffrage for women," she wrote to Mill. "And in the meantime there are evils which press much more hardly on women . . ." Inequalities of property rights and other hardships and evils she felt strongly must be removed. A woman must be "a person," and not the less if she is married, otherwise the harm even to her husband is infinite. But she feared that a certain sense of opposition between men and women might be roused, which would only delay reforms. Women's political power, however, should be "direct and open," not indirect. As for herself, she was conscious of having more administrative influence than if she were a borough returning two members to Parliament. And she felt that her work "off the stage" from the sick-room which she inhabited for so many years after the brief, brilliant days of work in the Crimea was more effective than anything else that she could do. She had, in fact, *no time* to work for votes for women.

But what was it that made this sick-room work so marvellously efficient? Not, surely, the halo that shone round the Lady of the Lamp, wonderful though that was. Such past glory by itself would not have availed to bring successive statesmen to her sofa, to be taught how to reform the sanitation of English barracks, so as to lessen, as reform did at once lessen, the frightful peace mortality among soldiers, to learn how to care for white troops in India, how to make or keep soldiers human by giving them wholesome reading and recreation. Even that experience did not by itself create in her the ability to organise the whole profession of nursing. What, then, was her power and what her work?

Florence Nightingale did not suffer fools gladly. Efficiency in man or woman she believed to be almost wholly a matter of will. What you do not know, you can find out; what you are not able to do, you can learn to do; what you perceive that you ought to be, you may become. Her own "passion for statistics" amazed her ablest friend. She collected them, and made others collect so that the truth about conditions and needed remedies might be known. It was often a long, laborious, thankless task, but it prepared the way for action. So also the need for research into the causes of tropical disease brought her mind to bear on germs and cultures, and therefore on the foundation of laboratories at Millbank. And as with the greatest questions so with the smallest. When her training-school had been established, the students' diaries were sent to her to see, and if one showed weakness in writing and spelling, she would suggest dictation lessons. What could be remedied should be remedied, for "man is man and master of his fate," and woman is in like case. Her faith was in solid work done with brains and hand, that were continually gaining fresh knowledge and skill, rather than in enactments. It was not legislation that created the nursing profession as we know it.

